# Variability in echocardiographic measurements of left ventricular function in septic shock patients

**DOI:** 10.1186/s12947-015-0015-6

**Published:** 2015-04-15

**Authors:** Lina De Geer, Anna Oscarsson, Jan Engvall

**Affiliations:** Department of Intensive Care and Department of Medical and Health Sciences, Linköping University, Linköping, Sweden; Department of Clinical Physiology and Department of Medical and Health Sciences, Linköping University, Linköping, Sweden

**Keywords:** Echocardiography, Heart failure, Intensive care, Interobserver reliability, Repeatability, Shock, Septic

## Abstract

**Background:**

Echocardiography is increasingly used for haemodynamic evaluation and titration of therapy in intensive care, warranting reliable and reproducible measurements. The aim of this study was to evaluate the observer dependence of echocardiographic findings of left ventricular (LV) diastolic and systolic dysfunction in patients with septic shock.

**Methods:**

Echocardiograms performed in 47 adult patients admitted with septic shock to a general intensive care unit (ICU) were independently evaluated by one cardiologist and one intensivist for the following signs: decreased diastolic tissue velocity of the base of the LV septum (é), increased early mitral inflow (E) to é ratio (E/é), decreased LV ejection fraction (EF) and decreased LV global longitudinal peak strain (GLPS). Diastolic dysfunction was defined as é <8.0 cm/s and/or E/é ≥15 and systolic dysfunction as EF <50% and/or GLPS > −15%. Ten randomly selected examinations were re-analysed two months later. Pearson’s *r* was used to test the correlation and Bland-Altman plots to assess the agreement between observers. Kappa statistics were used to test the consistency between readers and intraclass correlation coefficients (ICC) for inter- and intraobserver variability.

**Results:**

In 44 patients (94%), image quality was sufficient for echocardiographic measurements. The agreement between observers was moderate (k = 0.60 for é, k = 0.50 for E/é and k = 0.60 for EF) to good (k = 0.71 for GLPS). Pearson’s r was 0.76 for é, 0.85 for E/é, 0.78 for EF and 0.84 for GLPS (p < 0.001 for all four). The ICC between observers for é was very good (0.85; 95% confidence interval (CI) 0.73-0.92), good for E/é (0.70; 95% CI 0.45 – 0.84), very good for EF (0.87; 95% CI 0.77 – 0.93), excellent for GLPS (0.91; 95% CI 0.74 – 0.95), and very good for all measures repeated by one of the observers. On Bland-Altman analysis, the mean differences and 95% limits of agreement for é, E/é, EF and GLPS were −0.01 (0.04 – 0.07), 2.0 (−14.2 – 18.1), 0.86 (−16 – 14.3) and 0.04 (−5.04 – 5.12), respectively.

**Conclusions:**

Moderate observer-related differences in assessing LV dysfunction were seen. GLPS is the least user dependent and most reproducible echocardiographic measurement of LV function in septic shock.

**Electronic supplementary material:**

The online version of this article (doi:10.1186/s12947-015-0015-6) contains supplementary material, which is available to authorized users.

## Background

Cardiac dysfunction is a well-known complication of septic shock. It was first described using radionuclide cineangiography [1], and has been studied further since the introduction of echocardiography in intensive care clinical practice. Numerous studies have described diastolic as well as systolic dysfunction, or a combination of the two, in septic shock, using a variety of different echocardiographic parameters [2-6]. Furthermore, echocardiography is now increasingly used for haemodynamic monitoring and in titrating therapy in septic shock [7].

The frequency of cardiac dysfunction in septic shock as well as its impact on mortality has differed vastly between studies [2,3,5]. Differences in study size, inclusion criteria and in the prevalence of pre-existing heart-disease may have contributed to the varying results. In cardiology settings, a substantial interobserver variability in echocardiographic assessment has been described, especially in stress echocardiography [8-10]. In intensive care, echocardiographic assessment is often complicated by tachycardia, high levels of catecholamines and difficulties in image acquisition, not unlike that of stress echocardiography. However, the addition of observer dependency to the interpretation of the echocardiographic findings, and its impact on the differing results reported, has been scarcely studied [9,11,12].

In this study, we assess the reliability of echocardiographic parameters used to indicate left ventricular dysfunction in patients with septic shock. Our aim is to identify the least observer-dependent echocardiographic parameter describing diastolic and systolic left ventricular function.

## Methods

This study was approved by the Regional Ethical Review Board in Linköping, Sweden (Dnr 2012/233-31). When possible, informed consent was sought from patients at inclusion. Due to the observational nature of the study we were permitted to assume consent in patients who because of acute illness were unable to give their permission. In these cases, informed consent was obtained as soon as possible after recovery.

The dataset used for this study was originally collected for a study assessing speckle tracking echocardiography in septic shock patients [13]. Patients aged 18 years or older, admitted to the mixed non-cardiothoracic ICU of Linköping University Hospital, presenting with septic shock and with an expected ICU stay of 24 hours or longer, were screened for eligibility. In total, 50 patients were included from October 2012 to September 2014. Patients could be included only once. Patients who by the treating physician were not expected to survive longer than 24 hours, in whom intensive care treatment was partly withheld from admission, or who due to language barriers or mental inability were not expected to be able to give consent even after recovery, were excluded.

Septic shock was defined according to the Surviving Sepsis Campaign (SSC) criteria, and all patients were managed in compliance with the SSC guidelines for the treatment of septic shock [14]. Patients were considered to have a history of cardiac disease if they had prior or current ischaemic heart disease, cardiac surgery, hypertension or cardiac failure.

Transthoracic echocardiography was performed on the day of admission, all by an expert echocardiographer not involved in patient care (JE). A Vivid E9 ultrasound scanner was used, acquiring two-dimensional (2D) apical two-chamber, four-chamber and long axis views (2C, 4C and ALAX) of the left ventricle at a frame rate of >40 frames/sec. Images were analysed offline using dedicated software (EchoPac BT 112, GE Ultrasound, Horten, Norway), independently by two observers (LDG and JE). E-velocity was measured using pulsed wave (PW) Doppler in the mitral inflow at the tip of the valve. Diastolic tissue velocity of the base of the LV septum (é) was measured in the apical 4C view using PW tissue Doppler, and E/é ratios were calculated. Left ventricular (LV) volumes and ejection fraction (EF) were calculated using the modified biplanar Simpson’s method. Global longitudinal peak strain (GLPS) was calculated as the average speckle tracking strain from the 18 LV segments from the 2C, 4C and ALAX views (six segments per view in three different views). All echocardiographic studies were recorded over three consecutive cardiac cycles, independently of breathing cycles, and averaged. In patients with non-sinus rhythm measurements were collected and averaged over 5 – 10 heartbeats. Diastolic dysfunction was defined as E/é >15 and/or é <8 cm/s [15]. Systolic dysfunction was defined as EF <50% [16], and GLPS was considered decreased when > −15% [17]. A random sample of 10 examinations were re-analysed two months later by one of the observers (LDG) in order to study intra-observer repeatability.

Clinical data on co-morbidities and on ICU length of stay and outcome were all collected prospectively. To assess severity of illness, Simplified Acute Physiology Score 3 (SAPS 3) and Sequential Organ Failure Assessment (SOFA) score were calculated on admission.

### Statistical analysis

Data are presented as medians (lower quartile – upper quartile), numbers (percentages), and means with standard deviations (range), as appropriate. Inter-observer variability of echocardiographic parameters was determined by the intra-class correlation coefficient, Pearson’s correlation coefficient and Bland-Altman plots [18]. Reliability analyses using kappa statistics were performed to determine consistency between observers regarding the presence or absence of diastolic or systolic dysfunction. The kappa value for agreement was interpreted as follows: poor <0.20, fair, 0.21 – 0.40; moderate, 0.41 – 0.60; good, 0.61 – 0.80; and very good, 0–81 – 1.0 [19]. Intra-observer repeatability was calculated using intra-class correlation coefficient. All probability values are two-tailed and significance was set at p < 0.05. All statistical analyses were performed using IBM SPSS v22.0 (IBM Corp, Armonk, NY, USA).

## Results

Fifty patients in septic shock were included. Two patients died before echocardiography could be undertaken and in one patient, images were lost in the storage process. These three patients were excluded from the statistical analysis. In the remaining 47 patients, echocardiograms from the day of ICU admission were available. Twenty-three of the patients (49%) had previously known cardiac comorbidities, but there were no patients with severe valvular regurgitation, and none with endocarditis. A three-lead electrocardiogram (ECG) recorded for monitoring purposes was analysed regarding cardiac rhythm, but 12-lead ECG was not performed. Table 1 summarizes the main clinical characteristics of studied patients.

**Table 1:** **Baseline and echocardiographic characteristics of studied patients**

Number of pts, n	47
Male sex, n (%)	29 (62)
Age, median (IQR)	65 (57 – 74)
SAPS 3, median (IQR)	73 (45 – 84)
EMR, median (IQR)	60 (38 – 76)
SOFA day 1, median (IQR)	11 (9 – 12)
ICU LOS, days, median (IQR)	5 (2 – 12)
Mechanical ventilation at time of echocardiography, n (%)	35 (74)
Heart rate at time of echocardiography, median (IQR)	102 (87 – 112)
Atrial fibrillation at time of echocardiography, n (%)	13 (28)
Pre-existing cardiac disease, n (%)	23 (49)
Death in ICU, n (%)	10 (21)
Death within 30 days, n (%)	12 (26)
Death within 90 days, n (%)	14 (30)

Data are presented as medians (IQR) and numbers (%), as appropriate.

In three patients (6%), image quality was too poor to allow echocardiographic analysis. In the remaining 44 patients (94%), E, é, LVEF and GLPS could be assessed. Table 2 shows the diastolic echocardiographic parameters obtained by the two observers. Of the 18 LV segments, GLPS could be measured in, on average, 17 segments by both observers (range 13 – 18 vs range 12 – 18).

**Table 2:** **Left ventricular function on day 1**

	**Observer 1**	**Observer 2**
é cm/s	8.1 ± 3.0 (1.7 – 19.0)	9.0 ± 4.0 (2.9 – 22.0)
E/é	14.4 ± 11.7 (5.0 – 68.1)	11.4 ± 8.9 (1.0 – 52.3)
EF, %	49 ± 11 (17 – 76)	50 ± 12 (22 – 75)
GLPS, %	−16.6 ± 4.4 (−26.7 – (−3.1))	−16.6 ± 4.6 (−24.5 – (−3.9))

Means ± standard deviation (range) for diastolic and systolic function parameters measured by the two observers.

**Figure 1:**
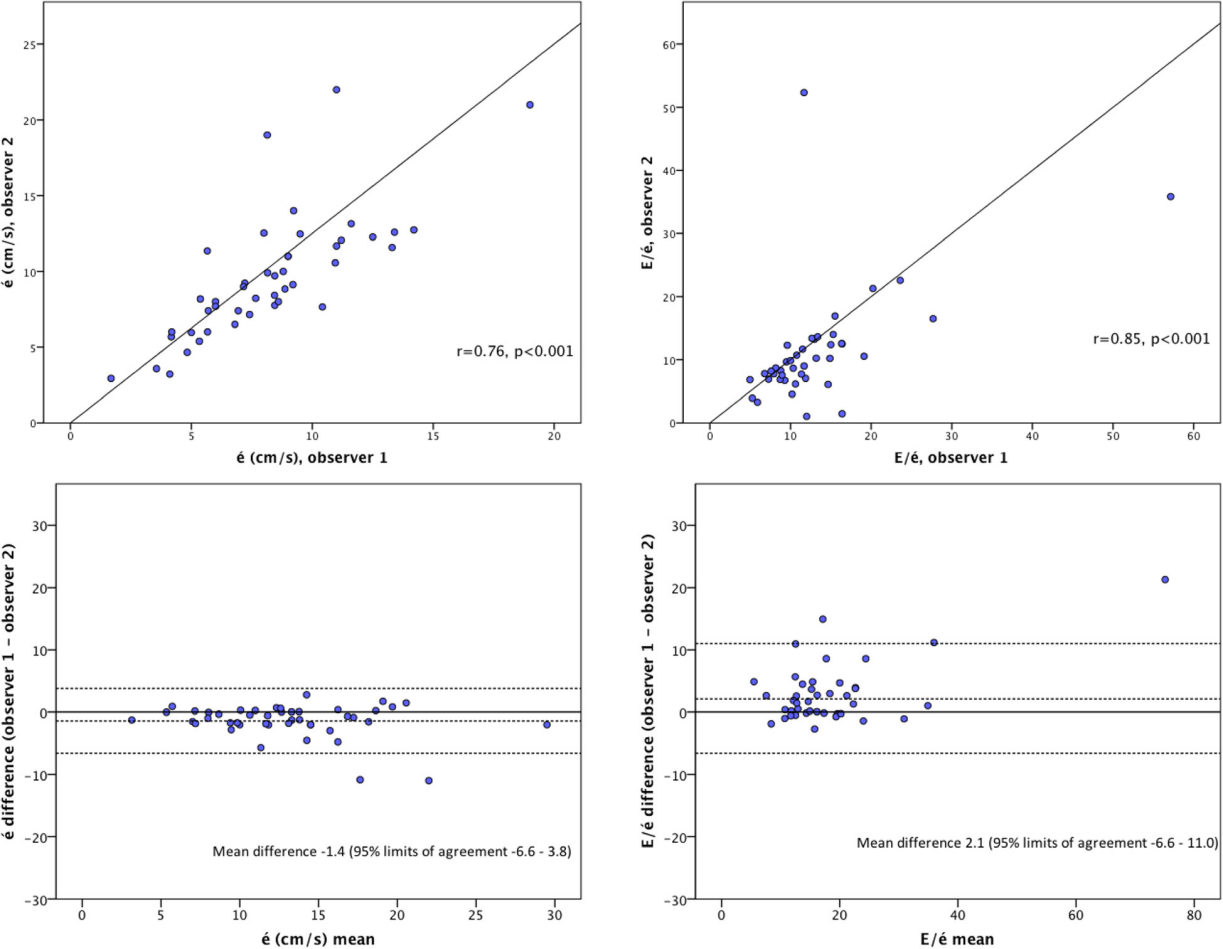
The correlation between and Bland-Altman analysis of é and E/é measured by two observers. Pearson’s correlation coefficient, mean measurements, differences and 95% limits of agreement (1.96 SD) between measurements by two observers.

The é measured by the two observers was 8.1 ± 3.0 and 9.0 ± 4.1, respectively (Table 2). On Pearson’s correlation test, r = 0.76 (p < 0.001, Figure 1), and the intraclass correlation was 0.85 (95% CI 0.20 – 0.73, Table 3). On Bland-Altman analysis of é measurements, the mean difference was 1.4 cm/s and the standard deviation (SD) 2.7 (Figure 1). Diastolic dysfunction defined as é <8.0 cm/s was identified in 21 (45%) and 18 (38%) patients, respectively. The observers agreed on diastolic dysfunction being present 15 patients (71% of those where at least one observer had indicated the presence of diastolic dysfunction). The inter-observer agreement was found to be kappa 0.60 (95% CI 0.36 – 0.83).

**Table 3:** **Repeatability of results**

	**é**		**E**/**é**		**LVEF**		**GLPS**	
	**ICC**	**p**	**ICC**	**p**	**ICC**	**p**	**ICC**	**p**
Interobserver	0.85 (0.73 – 0.92)	<0.001	0.70 (0.45 – 0.84)	<0.001	0.87 (0.77 – 0.93)	<0.001	0.91 (0.74 - 0.95)	<0.001
Intraobserver	0.91 (0.58 – 0.98)	0.002	0.95 (0.80 – 0.99)	<0.001	0.84 (0.75 – 0.90)	<0.001	0.89 (0.55-0.97)	0.002

Intraclass correlation coefficients (95% CI) and level of significance for the inter- and intraobserver variability of diastolic and systolic LV parameters.

The E/é ratio derived from measurements by the two observers was 14.4 ± 11.7 and 11.4 ± 8.9 (Table 1), r was 0.85 (p < 0.001, Figure 1) and the intraclass correlation coefficient 0.71 (95% CI 0.46 – 0.84, Table 2). The Bland-Altman plot in Figure 1 shows the mean difference being 2.1 (SD 8.25). When LV diastolic dysfunction was defined as E/é >15, the observers identified LV diastolic dysfunction in 11 (23%) and 6 (13%) patients, respectively, with agreement in 5 (45%), and the kappa was 0.50 (95% CI 0.19 – 0.80).

**Figure 2:**
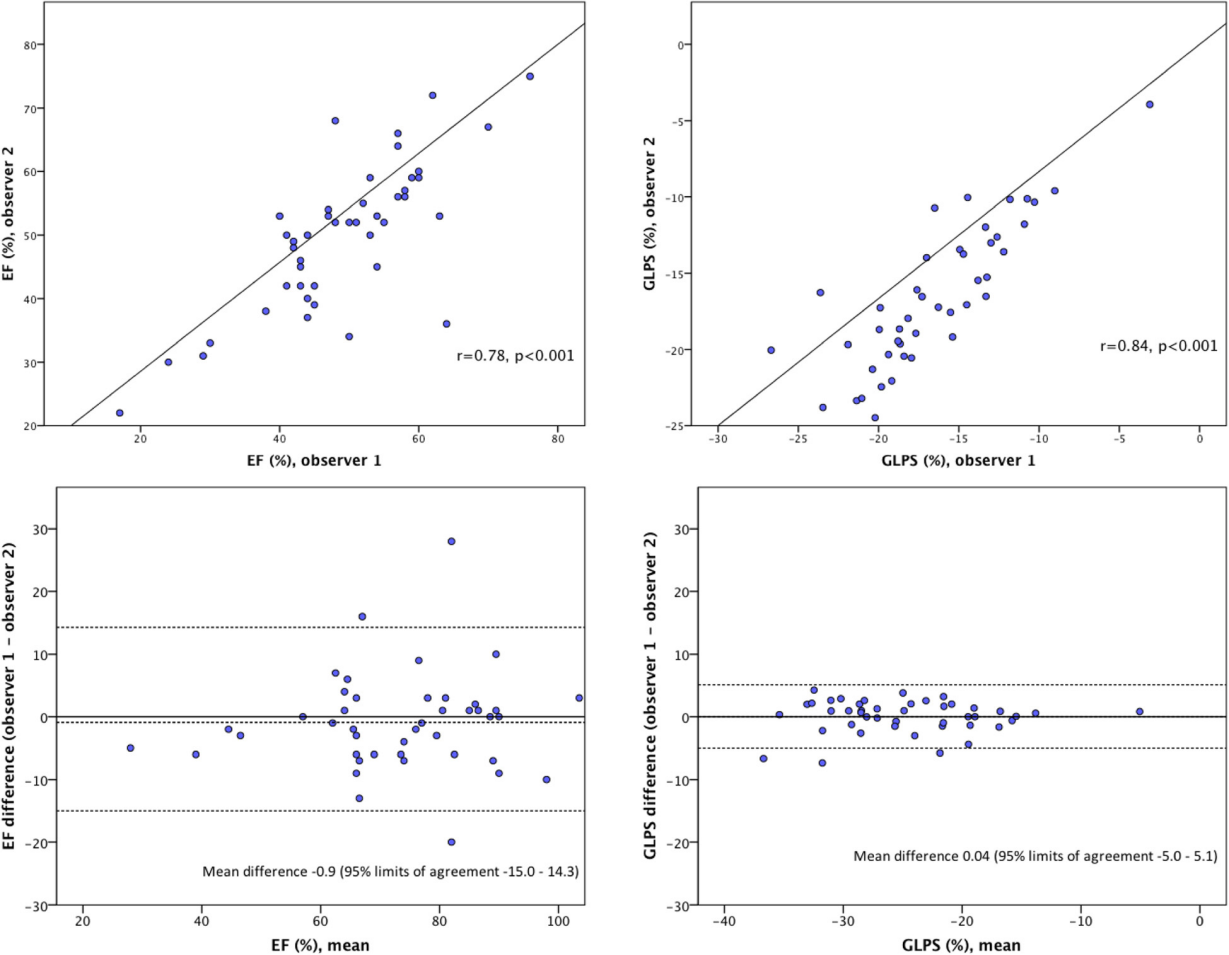
The correlation between and Bland-Altman analysis of EF and GLPS measured by two observers. Pearson’s correlation coefficient, mean measurements, differences and 95% limits of agreement (1.96 SD) between measurements by two observers.

EF measured by the two observers was 49 ± 11 and 50 ± 12, r = 0.78 (p < 0.001, Figure 2), the intraclass correlation coefficient was 0.87 (95% CI 0.77 – 0.93, Table 2) and the mean difference was 0.9% (SD 7.7, Figure 2). Decreased EF, defined as <50%, was considered present in 24 (51%) vs 21 (45%) patients. The observers agreed on EF being decreased in 18 patients (75%), yielding a kappa of 0.60 (95% CI 0.36 – 0.83).

**Figure 3:**
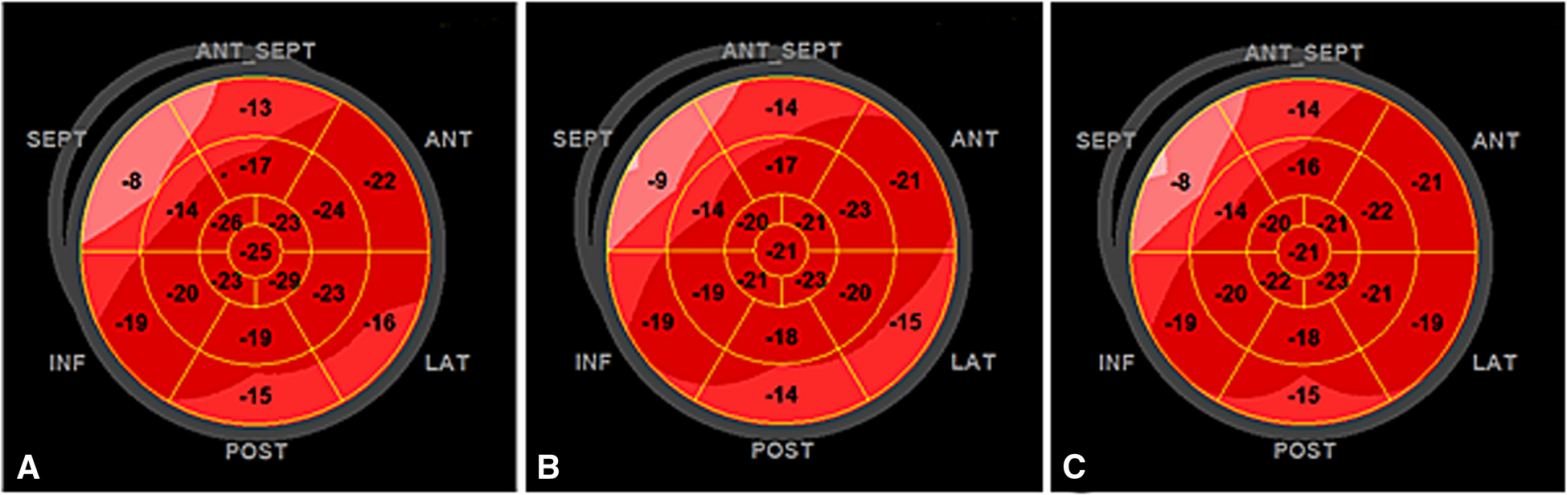
Variability of GLPS measurements. Segmental strain and GLPS of the left ventricle illustrated using automatic function imaging from three apical views and averaged for 18 segments; **A**. Measurements performed by observer 1, GLPS −17.9%; **B**. Measurements performed by observer 2, GLPS −20.0%; and **C**. Measurements re-assessed by observer 2, GLPS −18.4%.

The mean GLPS was −16.6 ± 4.4 and −16.6 ± 4.6, when measured by the two observers. The correlation coefficient (r) was 0.84 (p < 0.001, Figure 2) and the intraclass correlation coefficient was 0.91 (95% CI 0.84 – 0.95). An example of observed inter- and intraobserver reproducibility is illustrated in Figure 3 and is further highlighted in Additional file 1. The Bland-Altman plot in Figure 2 shows a mean difference of 0.04% (SD 2.6). The observers found GLPS decreased in 17 (36%) and 15 (32%) patients, respectively, agreeing in 13 (76%), and the inter-rater reliability was found to be kappa 0.71 (95% CI 0.49 – 0.92). Table 3 summarizes the repeatability of results, showing the intraclass correlation coefficients of inter- and intraobserver variability of LV function parameters studied.

Thirteen patients had atrial fibrillation when the echocardiographic examination was performed. When these were excluded from the analysis, Pearson’s correlation for é, E/é, EF and GLPS was 0.72, 0.80, 0.79 and 0.81, respectively (in all cases p < 0.001). ICC (95% CI) improved slightly to 0.83 (0.64 – 0.92), 0.88 (0.75 – 0.95), 0.88 (0.76 – 0.94) and 0.90 (0.79 – 0.95) for é, E/é, EF and GLPS. On Bland-Altman analyses, the mean differences and 95% limits of agreement (1.96 SD) for é, E/é, EF and GLPS were −1.8 (−7.8 – 4.2), 3.82 (−6.8 –14.5), −0.26 (−15.2 – 14.7) and 0.09 (−5.5 – 5.7), respectively.

## Discussion

In this study, we aimed at analysing the observer dependence of echocardiographic signs of cardiac dysfunction in patients with septic shock to establish the most robust, user independent parameter. Our findings suggest that there are moderate inter-individual differences regarding the measurement of diastolic and systolic parameters used to identify cardiac dysfunction in septic shock. GLPS is the least user-dependent and most reproducible parameter.

LV dysfunction in septic shock has been described with varying incidence. Impaired diastolic function with decreased é or increased E/é, as well as systolic dysfunction with decreased EF, has been reported in 20 – 60% of patients. A direct comparison between studies is complicated by differences in study size and in the prevalence of pre-existing cardiac disease, but also by the lack of true consensus on the definition of LV dysfunction in septic shock. In cardiology settings, the impact of observer dependence on echocardiographic measurements regarding diastolic as well as systolic parameters has been studied [8-10], but remarkably less so in the intensive care setting. Thus, to what extent the differing results regarding prevalence and implications on outcome in septic shock depends on the reading of the echocardiograms is unknown.

The echocardiographic measures to assess diastolic myocardial function, in this study é and E/é, reflect different aspects of LV filling, each with potential limitations. Furthermore, the measurements are manually obtained and therefore inherently subjective. Also, the accuracy and reproducibility of é and E/é is debated in patients with atrial fibrillation (AF), ischaemic heart disease or hypertrophic cardiomyopathy [20-22]. The prevalence of AF and other heart diseases in this study is in the same range as in other studies on septic shock patients [2,23]. In patients with AF, measurements were performed and averaged over a larger number of cardiac cycles. Still, our results indicate that atrial fibrillation hampers the consistency between measurements, possibly explaining some of the observed differences in previous studies.

The accuracy of é as well as E/é has been shown to be dependent on changes in volume loading and ventilator settings [24-26], possibly yielding different results even within the same echocardiographic examination and further highlighting the difficulties associated with the clinical intensive care situation. In heart failure patients, the observer concordance of diastolic measurements has been shown to be less than ideal [8]. Our results are in the same range as in this previous study, possibly reflecting similar difficulties in assessing diastolic function in septic shock patients as in those with heart failure. Interestingly, diastolic dysfunction in septic shock has, albeit with conflicting results, been shown to increase mortality [5,23]. Observer-related variability, such as shown here, may have contributed to the vastly differing results reported regarding diastolic function and its impact on mortality in septic shock patients.

For evaluating systolic LV function, we used the biplane Simpson method which is widely recommended and has been shown to reliably detect even small changes in EF in ischaemic heart disease [16,27]. One previous study has focused on the repeatability of LV systolic function parameters in intensive care patients in shock, the majority of which were in septic shock [11]. In this study inter- and intraobserver reproducibility was within a range that was considered acceptable, but the use of Simpson’s single plane method in addition to other measures of LV systolic function complicates direct comparison with our study. We found the agreement between observers to be moderate when defining EF as decreased if below 50%, in contrast to the very good correlation between observers regarding absolute values. This may illustrate the effect of an arbitrary cut-off, and may also be a reflection of the varying incidence and the unclear impact on mortality of decreased EF in septic shock, recently reported in a meta-analysis [28]. Interestingly, the presence of atrial fibrillation had little impact on the concordance of the results regarding systolic measurements.

In stress echocardiography a high degree of variability has been demonstrated even among expert observers [9]. A recent study has shown a much improved reproducibility of results in the interpretation of dobutamine stress echocardiography when using GLPS [10]. In line with this, our findings indicate a good reproducibility of GLPS in septic shock, with a small, scarcely clinically relevant, difference between observers and without obvious bias. There are indeed similarities between the context of stress echocardiography and that of echocardiography in septic shock patients, both situations being challenged by difficulties in image acquisition, high levels of endogenous and exogenous catecholamines in addition to tachycardia and arrhythmias. The superior reproducibility of GLPS in stress echocardiography, also shown here in septic shock, may reflect the previously described advantage of a semi-automated technique with less manual processing [29]. GLPS may thus represent an objective and possibly less user-dependent method than other echocardiographic measurements.

Our findings have clinical implications since echocardiography is used for the diagnosis of cardiac dysfunction in septic shock. Furthermore, echocardiography is increasingly recommended for circulatory monitoring in septic shock patients [7,30]. Since we found considerable interobserver variability in diastolic measurements, our results imply the need for some caution in using them to guide therapy in this group of patients. Much of the treatment recommended in septic shock predominantly targets the systolic function of the heart [14]. The good repeatability of systolic function parameters found here may further encourage the use of echocardiography as a monitoring tool. However, our results suggest that echocardiographic findings should be interpreted with some caution, and in relation to other laboratory and clinical data in the individual patient. Furthermore, GLPS has recently been shown to be a sensitive marker of LV dysfunction in septic shock [6,13,31]. This fact, in addition to the strong reliability and reproducibility of GLPS shown here, indicates a future clinical role of GLPS as a marker of LV function in septic shock.

### Limitations

The selection of patients has a heavy influence on the results of echocardiographic assessment. In this study, patients expected to survive less than 24 hours were excluded, and this may have contributed to the high percentage of successful acquisitions. LV function parameters used in this study are all well established in patients with cardiac disease, but there is no clear definition on cardiac dysfunction in septic shock. Neither is there any consensus on what degree of variability in measurements is acceptable in this group of patients, since this aspect of echocardiographic assessment has not been addressed before. Furthermore, the number of patients studied is limited and the dataset used was not originally collected for these analyses.

A strength of the study is the fact that images were collected by an expert-level echocardiographer. Thus, the focus of the study is on the repeatability of measurements, irrespective of image acquisition. Moreover, all echocardiographic analyses were performed independently and while blinded to patient identity and clinical background, and the samples used for comparison are therefor of equal size. Further strength lies in the systematic approach to assessing inter- as well as intraobserver reliability and reproducibility.

## Conclusion

We conclude that echocardiographic measurement of LV function is feasible and reproducible in the majority of septic shock patients. Because of the observer-related differences in the measurements used, results should be interpreted in relation to other clinical findings when used for guiding therapy. GLPS is the least user dependent and most reproducible echocardiographic finding of LV function in septic shock. GLPS may thus represent a useful tool in the evaluation of LV function in this group of patients.

## Additional file

Additional file 1:
**Reproducibility of GLPS measurements in a four chamber view of the left ventricle.** The given figures are based on an average from six segments of this particular view. From left to right: Measurements performed by observer 1, GLPS −19.8%; Measurements performed by observer 2, GLPS −18.2%; and measurements re-assessed by observer 2, GLPS −18.4%.

## Abbreviations

2D: Two-dimensional
2C: Two-chamber view
4C: Four-chamber view
AF: Atrial fibrillation
ALAX: Apical long-axis view
CI: Confidence interval
é: Early mitral tissue Doppler velocity
E/é: Early mitral inflow to early mitral tissue Doppler velocity
ECG: Electrocardiogram
EF: Ejection fraction
GLPS: Global longitudinal peak strain
ICU: Intensive care unit
IQR: Inter-quartile range
LV: Left ventricular
PW: Pulsed wave Doppler
SAPS3: Simplified acute physiology score 3
SD: Standard deviation
SOFA: Sequential organ failure assessment
SSC: Surviving Sepsis Campaign
